# Pollination in the Anthropocene: a Moth Can Learn Ozone-Altered Floral Blends

**DOI:** 10.1007/s10886-020-01211-4

**Published:** 2020-09-02

**Authors:** Brynn Cook, Alexander Haverkamp, Bill S. Hansson, T’ai Roulston, Manuel Lerdau, Markus Knaden

**Affiliations:** 1 Max Planck Institute for Chemical Ecology, Department of Evolutionary Neuroethology, Jena, Germany; 2 Department of Environmental Sciences and Blandy Experimental Farm, University of Virginia, Boyce, VA USA; 3 Present Address: Laboratory of Entomology, Wageningen University, Wageningen, The Netherlands; 4 Departments of Environmental Sciences and of Biology, University of Virginia, Charlottesville, VA USA

**Keywords:** Anthropocene, Insect olfaction, Pollution, Pollination, *Manduca sexta*

## Abstract

Insect pollination is essential to many unmanaged and agricultural systems and as such is a key element in food production. However, floral scents that pollinating insects rely on to locate host plants may be altered by atmospheric oxidants, such as ozone, potentially making these cues less attractive or unrecognizable to foraging insects and decreasing pollinator efficacy. We demonstrate that levels of tropospheric ozone commonly found in many rural areas are sufficient to disrupt the innate attraction of the tobacco hawkmoth *Manduca sexta* to the odor of one of its preferred flowers, *Nicotiana alata.* However, we further find that visual navigation together with associative learning can offset this disruption. Foraging moths that initially find an ozone-altered floral scent unattractive can target an artificial flower using visual cues and associate the ozone-altered floral blend with a nectar reward. The ability to learn ozone-altered floral odors may enable pollinators to maintain communication with their co-evolutionary partners and reduce the negative impacts that anthropogenically elevated oxidants may have on plant-pollinator systems.

**Electronic supplementary material:**

The online version of this article (10.1007/s10886-020-01211-4) contains supplementary material, which is available to authorized users.

## Introduction

Pollination is integral to maintaining diverse and healthy ecosystems (Kevan 1999), and it strongly contributes to global food production (Klein et al. 2007). The coevolutionary relationship between plants and their pollinators is maintained when plants emit signals that pollinating insects can detect and recognize as belonging to a host plant. These signals include visual cues, such as brightly colored flowers, and olfactory cues – i.e. floral scents (Kunze and Gumbert, 2001). Because the visual acuity of insect pollinators is limited to a resolution of centimeters to a few meters for most flowers (Kapustjansky et al., 2010) (but see Ohashi and Yahara (2002) for visual detection of flower patches over longer distances), smell is recognized as an important sensory modality guiding pollinators to flowers over long distances. Floral scents consist of an array of volatile organic compounds (VOCs) that are emitted from a flower and travel downwind, forming a “scent pathway” that can lead pollinators through the landscape to their host plant.

Animal pollination depends on plants producing signals that are maintained in the landscape and that pollinators can recognize, yet the composition of a floral scent of a given plant species may vary both spatially and temporally. Alterations in floral scent over evolutionary time scales, such as changes in scent due to modifications in genes coding for specific VOCs, have been shaped by the coevolutionary partnership between plants and pollinators (Dudareva and Pichersky 2000). However, variation in scent production can also occur outside of the paradigm of plant-pollinator coevolution and include variations at sub-evolutionary timescales. A pollinator will inevitably encounter variable floral scents over its lifetime even if it forages on just one plant species. In unpolluted environments, floral scents can vary due to differences both in emissions and in changes in the floral scent that occur post-emission as it moves through the atmosphere (Theis et al. 2007); (Raguso et al. 2003); (Muhlemann et al. 2014) and seasonal cycles as plants progress through different phenological states (e.g.(Desurmont et al. 2015; Theis et al. 2007). Furthermore, production of floral scents may vary across space: a population of one species in the landscape may have a different genetic expression for floral scent than another patch of the same species (Haverkamp et al. 2018; Knudsen et al. 2006), and environmental gradients such as soil moisture and nutrient load can also influence floral scent production (Majetic et al. 2009). Post-emission floral scent transformation also contributes to the variability of floral scents across a landscape: changes to wind speed, temperature, and turbulence all affect the concentration of a floral scent that the insect experiences and the probability of and frequency at which a pollinator encounters a floral scent (Murlis et al. 1992); (Finelli et al. 2000). Likewise, a pollinator’s location in the landscape and its distance from the emitting host plant will dictate the frequency and intensity of its encounters with floral scents (Visser 1986).

Given the variability in floral scents within the spatial and temporal foraging breadth of a single insect, pollinators must exhibit strategies to cope with floral scent variation. Pollinators could simply manifest a broad innate attraction to many floral compounds (Bisch-Knaden et al. 2018), such that a relatively stable subset of compounds provides a reliable cue in the midst of variation. If a broad innate recognition of cues does not suffice to maintain attraction to variable floral scents, pollinators can use another coping strategy: learning. Pollinators can modulate their preference for flowers based on their experience, so that an insect with no innate recognition for a specific floral compound or blend of compounds can associate that olfactory signal with a nectar reward while feeding at the flower (Wright and Schiestl 2009). Learning in this way leads to an increased repertoire of olfactory cues; hawkmoths readily forage on an innately less-preferred host plant after olfactory-association, but they maintain their innate recognition for preferred host flowers (Riffell et al. 2013). Learning may also assist pollinators in recognizing floral blends that have been modified as they move downwind of the plant; after learning an odor, honeybees can later recognize that same odor at a lower concentration (Bhagavan and Smith 1997; Pelz et al. 1997). Another type of learning even enables insects to recognize compounds they have no first-hand associative experience with; pollinators may generalize their experience with one compound to another compound, if both compounds have a similar chemical structure (Daly et al. 2001) or with one blend to another, if the blend compositions are not too different (Sprayberry 2020). By responding similarly to chemically related compounds, pollinators can follow floral scents that differ at production due to plant genetics or physiological conditions, or that differ because of chemical shifts in the floral scent as it travels away from the plant. In these ways both plasticity in behavior and innate recognition of compounds may work together to enable pollinators to cope with variable olfactory cues.

The conditions causing floral scent variation described above have existed over evolutionary time scales, and thus the composition and concentration of floral scents produced by plants has been subjected to selection pressures within the overall coevolutionary relationship between plant species and their primary pollinators (Dudareva and Pichersky 2000). In this manner, the ability of pollinators to find rewarding host plants despite variation in chemical signals has been subject to selection pressure. Today, however, many pollinators face landscapes with steeply increased floral scent variation as a result of anthropogenic interferences (Jurgens and Bischoff 2017). Following the industrial revolution, there has been a dramatic increase in the tropospheric load of atmospheric pollutants, including the oxidant species nitrate radical and ozone, and potentially hydroxyl radical (Hauglustaine and Brasseur 2001; Naik et al. 2013; Spivakovsky et al. 2000). All three of these highly reactive oxidants can react with the carbon-carbon double bonds that are commonly found in floral volatiles (Atkinson and Arey 2003; Baker et al. 2004). Because floral volatiles have different structures and thus react differently from each other with a given oxidant, oxidant pollution leads to both decreases in some key compounds and changes in the relative concentrations of individual compounds in a floral blend (Farre-Armengol et al. 2016; Lusebrink et al. 2015; McFrederick et al. 2008) – both of which may be important cues to foraging insects (Bruce et al. 2005). Moreover, when oxidants react with a VOC, they induce a series of reactions that can lead to the production of secondary compounds. Secondary compounds may themselves be long-lived VOCs, many of which are compounds that share little similarity to the parent VOCs, e.g. formaldehyde, acetone, and carbon monoxide (Lee et al. 2006; McFrederick et al. 2008)*.* While previous work finds that a pollinator can cope with ‘noise’ in a floral blend resulting from addition of non-target biological VOCs as well as anthropogenic VOCs (Riffell et al. 2014), it is unclear if pollinators can maintain attraction to floral scents that lose compounds at different rates due to oxidation.

Tropospheric oxidants thus have the potential to alter floral scent plumes and impede pollinators attempting to locate host plants. One oxidant, ozone, has increased from approximately 10ppbv or less in preindustrial times (Hauglustaine and Brasseur 2001) to current averages in North America of 20-45ppbv (Vingarzan 2004), with spikes as high as 120ppbv during summertime ozone events (Fiore et al. 2002; Vingarzan 2004). To continue using floral scents as cues in a world with elevated tropospheric ozone, pollinators must either hone in on non-reactive volatile compounds, or they must learn the succession of odors they encounter in the landscape, ranging from highly ozone-altered blends at distances far from the host plant, to blends that are unaltered at the flower. Current work has established that ozone-altered floral blends are less attractive than unaltered blends to a variety of insects including a bumblebee and two specialist herbivores (Farre-Armengol et al. 2016; Fuentes et al. 2016; Li et al. 2016). Chemical modelling studies have predicted that ozone will react with floral blends across landscapes (McFrederick et al. 2009; McFrederick et al. 2008) and that, as a result, insects will be less adept at locating their host plants in ozone-enriched environments (Fuentes et al. 2016). While these works have demonstrated the potential for ozone to alter floral blends and impede insect foraging, neither empirical nor computational studies have considered the ability of insects to learn to identify and respond to ozone-altered blends. Can the flexibility in cue recognition or the learning abilities of pollinators, established to help these insects thrive in variable landscapes, assist them in recognizing floral cues even as air pollution alters the integrity of those blends?

We test the ability of one nighttime pollinator, the hawkmoth *Manudca sexta*, to recognize and learn ozone-altered floral blends of one of its preferred host flowers, *Nicotiana alata*. While ozone is a significant oxidant at night, it typically has less oxidizing power than the other common nighttime oxidant, nitrate radical. Although both compounds react with carbon-carbon double bonds in floral volatiles, they do so through different mechanisms, resulting in different specific reaction products for nitrate radical versus ozone with floral blends. Unlike nitrate radical, however, ozone can be readily controlled in a laboratory setting, and we used it to examine the degree of behavioral plasticity a pollinator can demonstrate in response to an oxidant-altered floral blend.

After demonstrating that ozone substantially alters the odor profile of *N. alata* and renders it unattractive to naive moths, we develop an odor learning protocol for *M. sexta.* We consider two possible learning scenarios in which *M. sexta* could navigate to its host using odor cues, despite the alteration of the floral blend by ozone. First, we test whether *M. sexta* can learn to associate an initially unattractive ozone-altered floral blend with a sucrose reward. Second, we test whether a moth’s foraging experience in the presence of an unpolluted floral blend broadens the suite of cues used in host recognition. If so, after this experience, moths could find the floral blend attractive despite ozonation.

Further, we ran a separate learning experiment where the floral scent was decoupled from the sucrose reward. Assessing the moth’s ability to learn the ozone-altered blend decoupled from the reward is critical as a foraging pollinator would not have the ability to directly receive a reward while exposed to the ozone-altered floral scent: at the flower, floral blends have been exposed to ozone for a negligible amount of time, and it is only as the floral scent moves away from the source through a polluted atmosphere does it become altered (McFrederick et al. 2008).

Finally, we examine whether following and foraging on a pure plume alone is sufficient to enable moths to increase their preference for an ozonated plume, given their ability to generalize learned information and given some of the similarities between a floral plume mixed with just air vs. one that has been altered by ozone.

## Methods

To assess the response of *Manduca sexta* to ozone-mixed floral blends, we first analyzed the effect of ozone on the chemical composition of blends of *Nicotiana alata* flowers (for details on the GC-MS analyzes see Supplemental Methods). We next determined whether ozone-altered blends were less attractive than unaltered blends by presenting naïve male moths with both ozone-altered and non-altered blends in a windtunnel assay. After determining the response of naïve moths to ozone-altered blends, we next assessed their ability to learn the ozone-altered blend by luring the moths to feed at a visually attractive artificial flower while being exposed to ozone-altered blends, and subsequently testing whether or not this experience changed the moths’ preference for these blends.

**Fig. 1:**
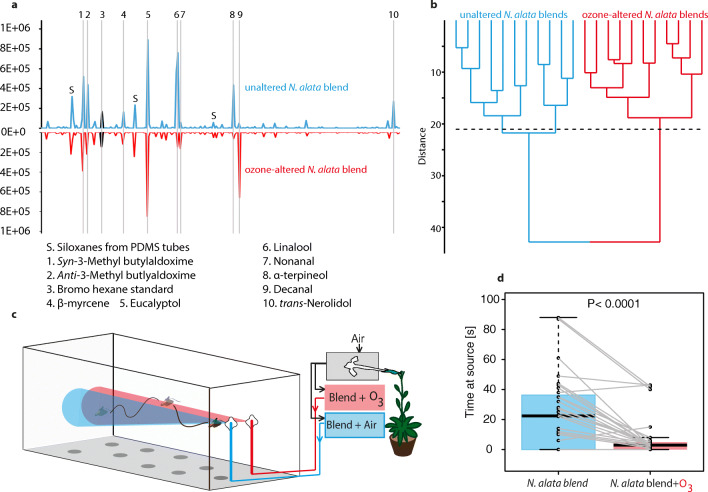
Hawkmoths prefer original over ozone-altered *N. alata* flower blends. A. Traces of unaltered and ozone-altered headspaces of *N. alata* flowers. Numbered peaks identified by the NIST library (R-match >90%) or by co-elution with synthetic compounds. B. Cluster analysis of 11 unaltered and ozone-altered flower headspaces. Terminal lines, replicate chemical analyses from both treatments. Analysis based on Ward’s algorithm and Euclidian similarity index using peak area values depicted in Table S1. Automatic truncation (dashed line) separates both treatments. C. Choice assay in wind tunnel (Plexiglas, LxHxW: 250x90x90cm) between two tubes emitting either unaltered or ozone-altered flower headspaces. D. Moths spend more time at the source emitting the unaltered floral scent (Wilcoxon signed rank test, *N* = 31, *p* < 0.0001)

*Floral Blends and Ozone-altered Blend Production* Plants in this study originate from an inbred line cultivated at the Max Planck Institute for Chemical Ecology, Jena, Germany since the year 2000. Odors of their flowers have been shown to be very attractive to the moths of our lab colony in wind tunnel assays (Haverkamp et al. 2016). Ozone-altered and unaltered blends were generated through two separate series of mixing bottles and released separately from a Teflon tube held upright in a metal cylinder in the wind tunnel (Fig. 1 and Fig. S1).

To generate floral blends, two one-day old flowers of *Nicotiana alata,* which had been grown under the same light and climate conditions as the moth*,* were placed in a screw-tight box with an inlet of clean air flowing at ~3 l/min, without removing them from the plant. The floral scent was then pumped out of the box and split into two 1 l/min flows. Each 1 l/min flow was directed into a 2 l airtight glass mixing bottle, where either air or ozone was added at a rate of 0.5 l/min. The mixture of floral volatiles with air or ozone was then further mixed through a series of three 1 l bottles before being released in the wind tunnel at a rate of 0.5 l/min. Immediately before entering the wind tunnel, the concentration of ozone in the altered floral scent was measured at 10 s intervals, with concentrations kept between 110-120 ppbv, a high but not uncommon pollution level during North American summers (Fiore et al. 2002). Ozone was produced using an ozone generator (Model 165, Thermo Environmental Instruments, Inc.) and ozone concentrations were measured with an ozone-analyzer (Model 49i and Model 49C, Thermo Scientific Inc). Floral blends and ozone-altered floral blends were collected with 5 mm long Polydimethylsiloxane (PDMS) tubes (inner diameter 1.5 mm, outer diameter 2.3 mm) that were placed in line with the volatile blends immediately before the floral scent entered the wind tunnel. PDMS tubes collected volatiles in both the ozonated and non-ozonated floral scent lines for 20 min: after this time, the PDMS tubes were immediately collected and either run immediately through a GC-MS analysis, or placed in a deep freezer (−20 °C) in preparation for running the samples through the GC-MS. Ozone was not removed (such as by MnO_2_ or by scrubbers) before floral volatile collection because preliminary tests and earlier studies (Fick et al. 2001) find that such scrubbers can affect the reaction of ozone with some floral volatiles, resulting in products that are not observed in the absence of these scrubbers.

*Analyses of Flower Volatiles* Following the scent collection in the wind tunnel, PDMS tubes were analyzed individually using a thermal desorption unit (TDU, Gerstel, Germany) coupled to a temperature-programmable vaporizing unit (CIS 4, Gerstel, Germany), which was linked to an Agilent 7890A gas chromatograph (Agilent Technologies, CA) running in splitless mode and connected to an Agilent 5975C mass spectrometer (electron impact mode, 70 eV, ion source: 230 °C, quadrupole: 150 °C, mass scan range: 33–350 u). We used a nonpolar column (HP-5 MS UI, 30 m length, 0.25 mm ID, 0.25 μm film thickness, J and W Scientific, USA) under constant helium flow of 1.1 ml/min. The TDU temperature raised from 30 °C to 200 °C at a rate of 100 °C/min and held for 5 min. Volatized compounds were trapped within the CIS 4 cooled injection system at −50 °C and subsequently injected into the GC. The GC oven was programmed to hold 40 °C for 3 min, to increase the temperature at 5 °C/min to 200 °C, then to increase temperature at 20 °C/min to 260 °C, which was kept for 15 min. Data obtained in Agilent software (.D format) were converted to NetCDF files for further deconvolution analysis in the open-source package software XCMS (Smith et al., 2006) implemented in R (R Core Team,^2014^). The XCMS deconvolution process consists of four steps: peak picking, peak grouping, and retention time correction, followed by a second peak grouping, a detailed description can be found in (http://masspec.scripps.edu/xcms/xcms.php). The chromatographic peak detection within 30 to 2100 s was processed by using the CentWave algorithm method with a maximum expected deviation of m/z values (ppm; part per million) = 30, peak width = c(3,50), and signal to noise ratio cutoff (snthresh) = 20.

Peak area values from GC analysis (Fig. 1A) were normalized to the sum of the values within the sample and finally compared by a cluster analysis tool in Past software (http://folk.uio.no/ohammer/past/) (Fig. 1B).

*Moth Preparation* Moths in this study originate from a long-term lab population that every few years becomes refreshed by individuals caught in Utah (USA). Moths were raised in a temperature and light controlled chamber (light:dark = 16:8 h, 70% relative humidity and 25 °C during the light phase, and 60% relative humidity and 20 °C during the dark phase) so that the moths experienced nighttime conditions during the day, and were active during normal working hours of the researchers. Three-day old naïve virgin male moths were used for all behavioral assays: moths were transported from their rearing chamber to the wind tunnel room in individual baskets and given at least an hour to acclimate to the wind tunnel conditions (25 °C, 70% relative humidity) before experiments.

*Choice Assays* Individual *M. sexta* were placed at one end of the wind tunnel and were mildly provoked to initiate flight. At the upwind end of the wind tunnel, two tubes emitted the headspace of a *Nicotiana* flower, with one tube emitting the floral headspace in scrubbed air, and the other emitting the floral headspace mixed with ozone (see above). Moths in this choice assay were given four minutes to forage on the scent choices presented in the wind tunnel (Fig. 1C). The amount of time spent investigating a scent with an extended proboscis was recorded as an indicator of their interest in feeding at the scent. For details on the stimulus handling see supplementary material and Fig. S1.

*Learning Floral Scents Protocol* Moth learning was tested in a series of different odor assays to gain better insights into the mechanism by which the moth could learn ozone-altered blends. Initially, a moth’s ability to learn scents was determined by training moths on the individual floral volatile racemic linalool, which in a wind tunnel assay alone has been shown to be insufficient to induce feeding behavior in naïve moths (Bisch-Knaden et al. 2018). 12 µl of 10^−3^ linalool in mineral oil per test was added to an airtight bottle on filter paper; air flowed through the bottle and into the chamber at a rate of 0.5 l/min. *M. sexta* were then trained on this linalool odor in a three-step learning process (Fig. S2A-C). First, a moth’s initial attraction to the odor was assessed by releasing the moth in the wind tunnel containing two inconspicuous tubes emitting linalool or air. The time the moths spent probing each tube with their proboscis during five minutes of flight was recorded. By subtracting the time each moth spent at the air source from the time it spent at the linalool source, we calculated the relative preference for linalool. After a fifteen-minute rest period, the same moth was returned to the wind tunnel that now contained a light blue paper ‘flower’ with 10 µl of 30% sucrose solution emitting 0.5 l/min of the linalool odor. Moths were given four minutes to forage on the ‘flower’. A moth was considered trained after foraging at the ‘flower’ for at least one minute. Trained moths were given another 15 min rest interval before being returned to the wind tunnel to repeat their initial air vs. linalool choice test (see above). An increased preference for linalool relative to air after the moths had foraged from a linalool-emitting paper flower would indicate that the moths had learned the odor in this assay.

**Fig. 2:**
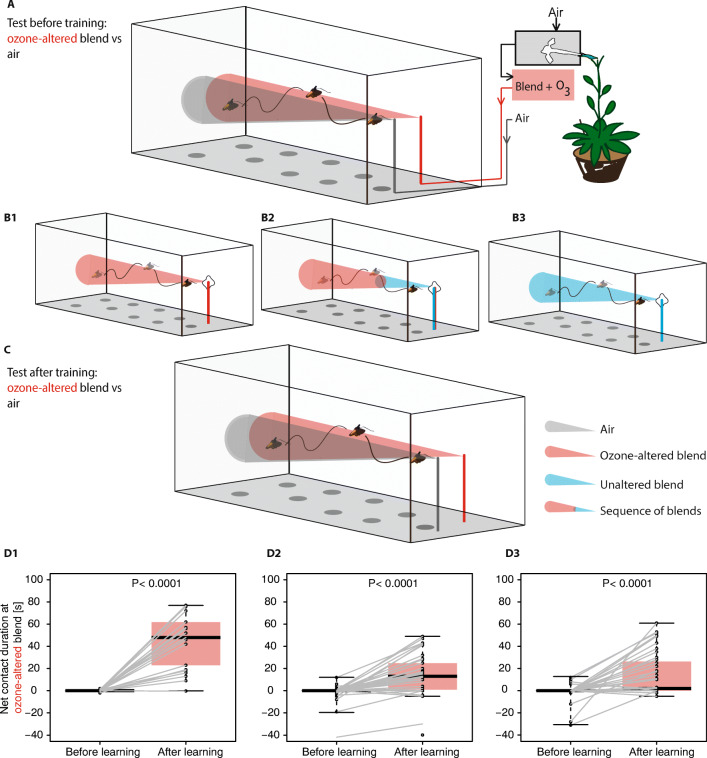
*Manduca* can learn to associate ozone-altered floral blends with a nectar reward. After initial testing (A), *Manduca* became sugar-rewarded at a visual cue emitting either an ozone-altered blend (B_1_), a blend that switches from ozone-altered to non-altered shortly before moths reach the flower (B_2_), or an unaltered blend (B_3_) and were tested again (C). D_1–3_. Moths prefer ozonated floral scent vs. clean air after but not before training in all training situations. Net contact duration at ozone-altered blend [s], time at ozone-altered floral scent minus time at clean air source [s] (Wilcoxon signed rank test, B1, *N* = 22, C1, *N* = 45, D1, *N* = 48)

*Learning Ozone-Altered Floral Scents* With a learning system established, we proceeded to test *M. sexta*’s ability to learn ozone-altered flower blends. Following the same three-step learning procedure as for linalool, we tested whether *M. sexta*’s preference for ozone-altered floral blends would increase after experiencing the scent paired with a sucrose reward. Firstly, male *M. sexta*’s initial preference for ozone-altered flower blend vs air, emitted from inconspicuous tubes (Fig. 2A), was tested to give a baseline attraction for the ozone-altered scent. Next, the moths were trained on the ozone-altered scent—moths were considered trained after they had foraged from a conspicuous artificial flower emitting the ozone altered flower blend and with the sucrose reward (10 µl of 30% sucrose) (Fig. 2B1). Following this training, the initial assay was repeated, with the now-trained moths able to investigate either the ozone-altered floral blend or air emitted from the inconspicuous tubes (Fig. 2C).

*Learning Ozone-altered Scents with Scent and Reward Decoupled* In an actual foraging environment, a moth would not have the opportunity to feed while being exposed to the ozone-altered plume because it is only as the plume moves downwind of the flower that it mixes with, and is altered by, ozone. To determine if moths could learn the ozone-altered plume decoupled from the sucrose reward, we altered the ‘training phase’ of our learning assay so that *M. sexta* were given just one minute to fly towards an artificial flower emitting an ozone-altered scent at an increased flow of 1 l/min. However, when the moth approached the flower to feed, with its proboscis extended within ~20 cm from the artificial flower, the ozone-altered scent was switched to an unaltered floral scent; thus only the unaltered floral scent was emitted when the moth fed at the sucrose reward (Fig. 2B2). The switch between the ozonated and pure odors was accomplished by a manual switch—as the moth crossed a 20 cm line marked in the wind tunnel with tape, the researcher pressed a button to switch the flow (controlled through two flow meters) from the ozonated to unozonated floral blends, which had been generated through the same series of bottles described above. When a given floral blend was not flowing into the wind tunnel, it was run through a series of charcoal scrubbers and released into the room external to the wind tunnel.

To determine if moths responded to a sequence of two odors we further tested the moth’s ability to learn a two-scent sequence in a situation in which linalool (a scent not innately-attractive to male moths (Riffell et al. 2009) led to a rewarding artificial flower emitting 2-phenylethanol (an innately-attractive scent) (S4).

*Generalizing Between Learned and Novel Scents* In a final experiment we trained the moths under otherwise identical conditions to the previously described learning assays, but had only the original blend present during the training phase (i.e., the moth never experienced the ozone-altered scent during the training phase) and tested whether experiencing the unaltered blend while feeding would affect their response to the altered flower blend afterward (Fig. 2B3).

## Results

*Ozonated Blends are Less Attractive to Hawkmoths* We first tested whether ozone-treated blends differ chemically from untreated floral blends. Ozone altered the emitted floral blend in three ways: it decreased the amount of several primary floral volatiles, changed relative ratios of primary floral volatile compounds, and created new “secondary” floral volatiles (Fig. 1A) resulting in a blend that differed from the unaltered one (Fig. 1B).

We next tested the moths’ innate preferences for floral blends that were either ozone-altered or not in a binary choice assay in a wind tunnel (Fig. 1C, for details on the stimulus handling see supplementary material and Fig. S1). In the wind tunnel, individual naïve *M. sexta* foraged on two artificial flowers, one exuding the ozone-altered, and the other the unaltered *N. alata* blend. While in preliminary tests ozone alone neither repelled nor attracted moths in the wind tunnel (Fig. S2), moths spent less time foraging on the floral blend mixed with ozone compared to the unaltered floral blend (Fig. 1D).

*Hawkmoths can Associate Ozone-altered Floral Blend with a Reward* Naïve male *M. sexta* moths found floral blends more attractive after training as assessed in a three -step learning assay (Fig. 2A-C). In the case where moths were tested, trained, and re-assessed for their interest in the ozone-altered plumes, we found that moths spent strikingly more time investigating the ozone-altered floral scent (probing with extended proboscis) after they were trained to it (Fig. 2D_1_, for learning of an innately non-attractive single odor under these conditions, see Fig. S3D_1_).

*Coping with Ozone-altered Odors in Natural Situation* In an actual foraging environment, a pollinator would not directly receive a reward while exposed to the ozone-altered plume: at the flower, floral blends have been exposed to ozone for a negligible amount of time, and it is only as the plume moves away from the source through a polluted atmosphere does it become altered (McFrederick et al. 2008). Can moths learn that ozone-altered floral blends lead to rewarding flowers, even when they only receive a floral reward while experiencing the non-altered plume? Although it is difficult to fully simulate an environment where foraging moths experience a progressively less ozone-altered plume as they approach the flower, we tested the moth in a situation where it first had to follow an ozone-altered plume, but finally was rewarded at a non-ozonated plume. To do so, we kept the pre- and post-training test phases as before, but modified our training phase so that the moth flew towards the artificial flower emitting the ozone-altered plume until the moth’s extended proboscis was only 20 cm away from the source. At that point, we switched the scent of the artificial flower to the unaltered plume and permitted the moth to feed (Fig. 2B_2_). Although the moths were never rewarded in the presence of an ozone-altered plume, moths still spent significantly more time at the ozone-altered plume after the training procedure (Fig. 2D_2_).

We next tested the moth’s ability to learn a two-scent sequence in a situation in which linalool (a scent not innately-attractive to male moths (Riffell et al. 2009) led to a rewarding artificial flower emitting 2-phenylethanol (an innately-attractive scent). We found that moths trained in this situation did not become attracted to linalool in a subsequent test (Fig. S4). This was not due to an inability to associate linalool with a reward, as they learned it easily when presented in isolation at the artificial flower (Fig. S3D_1_). This result suggests that *M. sexta* does not readily learn to associate two dissimilar blends in a sequence. The more parsimonious explanation is that the moths in our experiment recognized similarities between the ozone-altered and unaltered floral blends after experience.

To further test whether moths were able to generalize from the unaltered blend to the related ozone-altered blend after foraging, we ran a third learning trial (Fig. 2B_3_). For this experiment, moths were rewarded at the original unaltered floral blend (i.e. they did not experience the ozone-altered blend at all in the training phase); when these same moths were later presented with the ozone-altered blend they responded positively to it (Fig. 2D_3_). We conclude that experiencing the original unaltered plume with the nectar reward led moths to generalize their attraction from the pure plume to the ozonated plume. Experience with the unaltered blend, hence, could reinforce the attraction of innately neutral compounds that are not susceptible to degradation by ozone and later guide the moth also to the ozone-altered blend.

## Discussion

While oxidizing agents such as ozone have always been present in the atmosphere, the accelerated production of these pollutants following the industrial revolution has introduced a potential risk to pollination. We found that ozone significantly altered the blend of floral volatiles relative to an air-mixed plume (Fig. 1A and B). Our study does not quantify the impacts of ozone on the floral plume by documenting the decrease of individual volatiles after exposure to ozone: however, our results generally align with existing real-time volatile analysis and simulation and empirical studies (Farre-Armengol et al. 2016; McFrederick et al. 2009; McFrederick et al. 2008) in finding that ozone significantly alters a floral blend primarily by reducing individual volatiles. Further experiments that directly correlate ozone mixing times with distance and using real-time volatile analysis (as in Farre-Armengol et al. 2016) will be critical to understand the distances at which given ozone-levels interfere with floral blend recognition for a given pollinator. In our study, we simply found that ozone not only significantly altered the plume, but also made that plume less attractive for the hawkmoth *M. sexta* (Fig. 1C and D). This finding is congruent with results from naïve bumblebees (Farre-Armengol et al. 2016).

However, *M. sexta* has been shown to increase its preference to innately less attractive flowers after feeding on them (Riffell et al. 2008). Furthermore, moths can even become attracted to non-attractive scents after the scent is paired with an attractive visual cue (Goyret et al. 2007). Corresponding to that, we found that learning might help the moth in coping with the innately non-attractive ozonated floral blend: *M. sexta* spent more time attempting to forage at a source emitting an ozone-altered blend after experiencing this blend paired with a sucrose reward. This increased foraging time was not caused simply by an increased eagerness to forage after successful feeding: moths exhibited no significant increase in the foraging time when tested with an odor that differed from the training odor (Fig. S3D2). Hence, we conclude that *Manduca* can learn to associate an ozone-altered floral scent with a nectar reward, either by direct association of the altered-floral scent with reward (Fig. 2 D_1_), or when the ozonated blend is decoupled from the sucrose reward Fig. 2D_3_) or even when only the chemically similar but unaltered blend is presented with the reward (Fig. 2D_3_).

During training for these experiments, the moths fed in the presence of both a conspicuous artificial flower (visual cue) and an inconspicuous tube delivering the odor cue. Although this scent tube, which was present during testing, could have become part of the learned stimulus and contributed to the later response of the moths, there is strong evidence against this: in the consecutive cue experiment when moths experienced environmental linalool followed by 2-phenyl ethanol while feeding at the artificial flower, they did not respond positively when tested on linalool emitted from the same tubes.

There are two immediately apparent possible explanations for the moth’s learned response to the ozonated blend decoupled from the reward: 1) One explanation is that the moth learned that a sequence of two distinct scents led to a reward, a learning technique achievable by honeybees (Hussaini et al. 2007), or 2) experience at the pure floral scent broadened the perceived overlap in attractive components between the unaltered and the ozone-altered floral scent. However, moths that were trained with a sequence of linalool and 2-phenylethanol (with only the latter odor being present while the moth was rewarded) did not increase their preference to linalool (Fig. S4). At least in this scenario, *M. sexta* does not learn to associate two dissimilar blends in a sequence. The more parsimonious explanation is that the moths in our experiment recognized similarities between the ozone-altered and unaltered floral blends after experience.

This recognition of similarities between the blends post-training was further supported by the moth’s increased attraction to ozone-altered floral scents after foraging on only the original blend. We conclude that experiencing the original unaltered floral scent with the nectar reward led moths to generalize their attraction from the pure floral scent to the ozonated floral scent. Experience with the unaltered blend, hence, could reinforce the attraction of innately neutral compounds that are not susceptible to degradation by ozone and later guide the moth also to the ozone-altered blend. Likewise, it may be ascribed to a recognition of similarities between the ozonated and original blend after reinforcement through association.

Moths might already face similar challenges posed by floral blends changing over a spatial gradient in their natural environment as odor-blends are altered not only by pollutants but by natural occurring biotic and abiotic factors. To cope with this *M. sexta* has been shown to use particular neuronal coding mechanisms to preserve the identity of an attractive blend (Riffell et al. 2009). Our results suggest that these neuronal codes can be modified through learning and enable the moth to generalize between innately attractive and modified flower odor blends.

Although learning enables *M. sexta* to recognize an ozonated blend in a manner that it may be able to employ in the field, learning as a means of mitigating effects of anthropogenically-induced blend perturbation is not without limits. To begin, ozone is just one air pollutant that has increased as a result of anthropogenically driven fossil fuel combustion: nitrate radical, which peaks at night, has also risen due to increased nitrogen oxides in the atmosphere. In this study, ozone was used as a proxy for nitrate radical, but while both ozone and nitrate radical oxidize alkenes, they do so in a different manner. Contrary to ozone nitrate radical can also abstract hydrogens from C-H and even O-H bonds (Atkinson and Arey 2003; Baker et al. 2004), making it a more reactive oxidant than ozone. Furthermore, not all pollinators may have the same learning capability as *M. sexta.* As a crepuscular forager, *M. sexta* readily uses visual cues during foraging (Raguso and Willis 2002; Stockl et al. 2017); in our training paradigm, this partial reliance on visual cues attracted moths to forage on artificial flowers that emitted the non-attractive, ozone-altered blend. Because blends become more degraded as they move away from flowers, the odor floral scent that occurs beyond the visual range of a flower could be very different from the original one. An original scent may attract a pollinator within the visual range of a flower, and then both vision and olfaction synergize in guiding the pollinator to the flower (Kulahci et al. 2008). However, when floral scents become too degraded, moths may have to get within sight of the flower by chance, which could reduce pollination efficiency. Pollinators that rely less on visual cues might be even more perniciously affected, as they would not become at first attracted by visual cues and thereupon learn the ozone-altered floral scents or reinforce the pure floral scent. Additionally, *M. sexta* appears to innately restrict its foraging range to flowers of the so called “hawkmoth-pollination syndrome” all of which produce floral bouquets with similar compounds, but that differ in exact composition (Haverkamp et al. 2016; Riffell et al. 2013). Hence the ability to generalize within a syndrome may be an important aspect of the foraging behavior of *M. sexta*. In contrast, pollination systems in which pollinators become attracted to only a few unusual floral volatiles e.g. sexually deceptive orchids that attract pollinators by emitting the pollinators’ sex pheromones (Schiestl et al. 2003), could be more detrimentally affected if ozone reacts with their key attractive volatiles. We therefore do not claim that increasing ozone levels will not cause any pollination-related complications; rather, the data presented here suggest that learning by pollinators may mitigate such complications in some cases and that future studies should explicitly consider learning when searching for pollution’s impacts on pollination.

In summary, learning can provide one means by which pollinators can still use oxidized floral blends, but the importance of learning as a means of coping with polluted blends in the field remains to be tested. Learning capabilities are likely to be highly variable among species, and, hence, elevated tropospheric oxidants still pose a potentially serious threat to foraging pollinators. Multiple studies have reported declines in insect abundance and pollinator health in regions across the globe (Potts et al. 2010; Kluser and Peduzzi 2007; Fox et al. 2013; Hallman et al. 2018), and various anthropogenic drivers have been implicated in this decline. Anthropogenically elevated air pollution could be another stressor contributing to overall global insect declines. Future work is needed to assess the real threat of oxidants on foraging insects, and such work must consider pollinators as agents capable of plastic behavioral responses in the field.

## Electronic supplementary material

ESM 1 (DOCX 460 kb)
