# Supplementary material for: Pollination in the Anthropocene: a Moth Can Learn Ozone-Altered Floral Blends
Source: J Chem Ecol. 2020 Sep 2;46(10):987–96. doi: 10.1007/s10886-020-01211-4 (PMC7547994; doi:10.1007/s10886-020-01211-4)
Supplement: Supplementary file 1 — (DOCX 460 kb) [file 10886_2020_1211_MOESM1_ESM.docx]

Supplementary Material

*Floral blends and ozone-altered blend production.*

Ozone-altered and unaltered blends were generated through two separate series of mixing bottles and released separately from a Teflon tube held upright in a metal cylinder in the wind tunnel (Fig. S1).


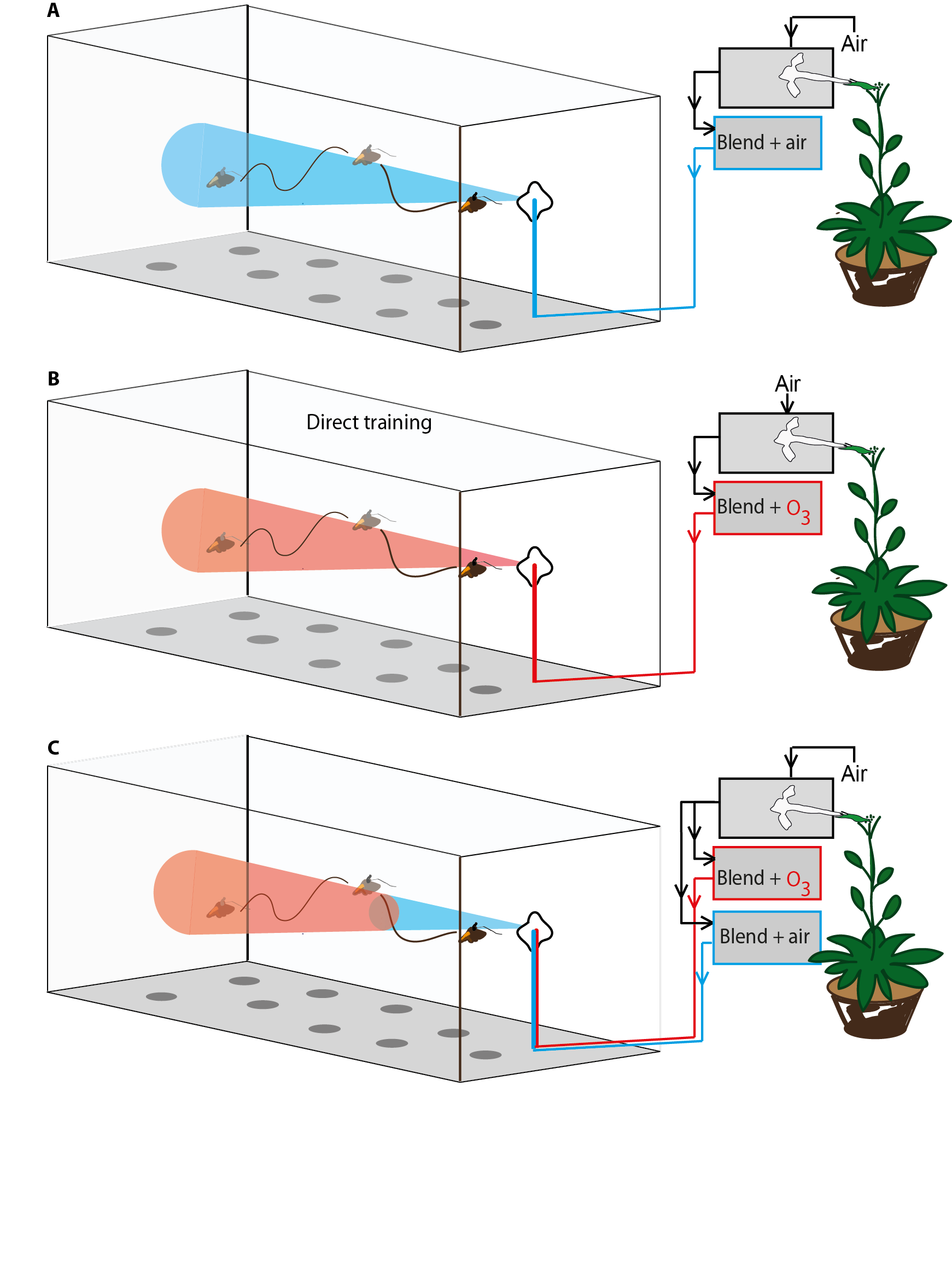


Fig.S1. Schematic of preparation of floral blends for wind tunnel experiments. A. Preparation of original *N. alata* blend. B. Preparation of ozone-altered *N. alata* blend. C. Preparation of *N. alata* blend that can be switched from original to ozone-altered when moth draws near the artificial flower. For details see text below.

Fig.S2. Moths neither prefer nor avoid an ozone source in the absence of floral odors. Lines connect times spent by individual moths at either source (Wilcoxon signed-rank test, N=36, p > 0.05). For details of the choice assay see Fig. 1 in manuscript.

*Learning floral scents in the wind tunnel*

To test, whether moths would be able to learn ozonated flower odors, we first established that moths would be able to associate an odor with a sugar reward in our wind tunnel assay (Fig.S2)


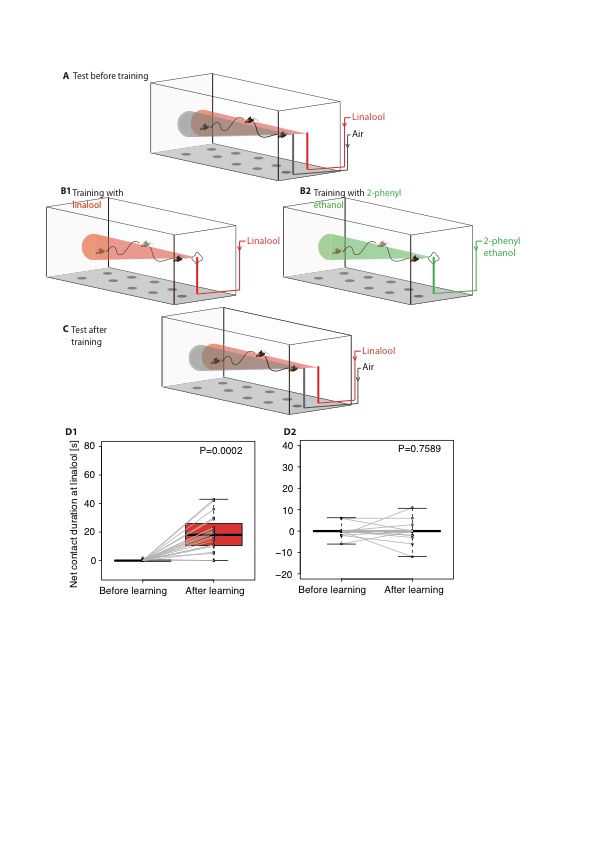


Fig.S3. Learning of a monomolecular odor. A. Test of the moth’s innated preference for linalool vs air. Bi. Training where the moth becomes sugar-rewarded at a visual cue emitting linalool. Bii. Training where the moth becomes sugar rewarded at a visual cue emitting another odor (2-phenyl ethanol). C. Test of the moth’s preference for linalool after it has been trained to linalool or 2-phenyl ethanol. Di. Preference for linalool increases after training with linalool (Wilcoxon signed-rank test, N=20, p < 0.01) but not after training with 2-phenyl ethanol (Wilcoxon signed-rank test, N=24, p > 0.05). Net contact duration at ozone-altered blend [s], time at linalool source minus time at clean air source [s]

*Behavioral effects of ozone*

To test whether the reduced attraction to ozonated flower blends was due to an aversion towards ozone, we tested, whether moths would treat an air source and a source emitting air enriched with ozone (110-120 pbbv) differentially (Fig. S3).

Moths, indeed, learned the association of linalool with sugar reward Fig. S2Di). To ensure that moths had learned linalool and were not merely more responsive to any scent presented after foraging, we switched the training compound to 2-phenyl-ethanol, and found that after this training moths did not increase the time spent investigating a linalool plume (Fig.S2Dii).


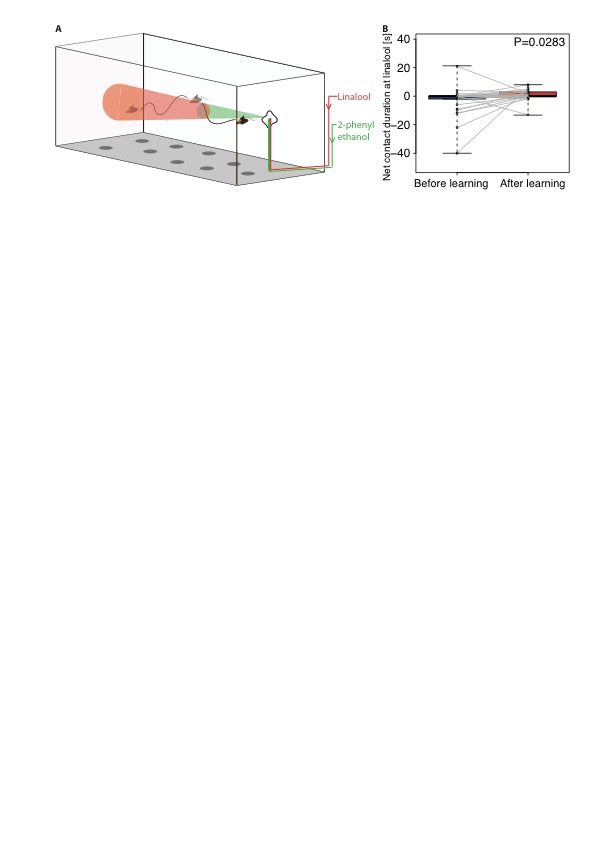
 Fig.S4. Moths do not learn a linalool plume when they follow this plume but become rewarded with another odor. Before learning: moths tested with linalool vs clean air when they had not experienced linalool before; after learning, the same moths after they were allowed to follow a linalool plume but then foraged on a feeder that emitted 2-phenyl ethanol (Wilcoxon signed-rank test, N=30, p > 0.05). Corrected time at linalool: for each moth, before and after the training preference was calculated as time at air source minus time at linalool source.
